# Academical Proceedings, National and Foreign

**Published:** 1822-06

**Authors:** 


### ACADEMICAL PROCEEDINGS,

### NATIONAL AND FOREIGN.

### 1. ROYAL INSTITUTE OF FRANCE.

AT the general meeting of the Academy of Sciences, held in April last, the gold medal, value 3000 francs, was adjudged to Professor Oersted, of Copenhagen, for his brilliant discovery of the action of the voltaic pile on the magnetic needle.

At this same meeting, Messrs. Desmoulins and Jules Cloquet received a gold medal, for their respective memoirs on subjects connected with Experimentals Physiology: the first being entitled "Recherches, anatomiques et physiologiques, sur le Système Nerveux dans les Poissons;" the second, "sur les Calculs Urinaires."

We understand that M. Cloquet has described, in his memoir, upwards of six thousand specimens of calculi, including every possible variety of form and constitution under which those concretions have been found; indicating, at the same time, various means through which nature itself often endeavours to get relieved of their presence,—such as their spontaneous solution, or rupture; and the decomposition of the animal part. His essay is remarkable for the number of experiments it details respecting the possibility of circulating within the bladder a large quantity of water, to the great and constant relief of the patient; a method first recommended by Dr. Arnott in this country, to whose invention, we trust, M. Cloquet has done full justice.

The Prize Questions, for 1823, connected with medical science, and proposed by this learned body, are the following:

1. *Prix de Physique*, to be adjudged at the general meeting of 1823.—*Determiner par des experiences precises quelles sont les causes, soit chirurgiques, soit physiologiques, de la chaleur animale.*

2. *Prix de Physiologie Experimentale*, to be adjudged at the same general meeting.—The subject is left to the option of the concurrents.

At the ordinary meeting of the 25th February, Dr. Desmoulins read a paper on the Geographical Distribution of Vertebrated Animals; from which it appears that this distribution bears no relation to the degree of temperature of the region they live in.

A memoir was read, at two meetings in March, by Dr. Flourens, on the Nerves; some parts of which are in direct contradiction with the observations of Mr. Charles Bell.

Extracts of a work on the extensive family of the *Arthrodices*, comprised in the genus *Conferves* of Linnæus, were read by M. Bory de Saint Vincent. This class of microscopic beings form the connecting link between the *conferves*, which are vegetable bodies, and the *vermes infusorii*, belonging to the animal kingdom.

### 2. ROYAL ACADEMY OF MEDICINE OF PARIS.

General meeting of the 26th of February.—A boy, five years of age, was presented to the Academy, as one of the most remarkable instances of obesity. He can scarcely walk. He is short of stature, and weighs one hundred pounds.

M. Beclard read an account of the malady to which M. Mazet fell a victim at Barcelona. The account is drawn up by Dr. Bailly, who was one of the commission; and has been ordered for publication.

### 3. SOCIETY OF MEDICINE, SURGERY, AND PHARMACY, OF THE DEPARTMENT OF THE EURE.

A prize question for a gold medal, value two hundred francs, has been proposed by this Society, to be adjudged in January 1823:

"To determine the different species of hydrorachitis; point out its causes and varieties, according to the age, the characteristic signs, the treatment, and the organic alterations observed in the parts which constitute the seat of the disease."

### 4. THE ROYAL SOCIETY OF MEDICINE OF BOURDEAUX.

Among the prize questions proposed by this Society, we have remarked the two following, as immediately connected with the practice of medicine.

A. To determine the nature, differences, causes, symptoms, and treatment, of the disease commonly called Œdema of the Lungs.

B. What are the consequences of a too rapid growth. What are the means of checking the progress of it, if found to be hurtful to the health of the individual; and of remedying any bad effect already produced?

### 5. SOCIETY OF PRACTICAL MEDICINE AT ST. PETERSBURGH.

This Society was founded in 1819, with a view to promote the advancement of medical science, of reciprocally communicating every useful medical observation among its members, and of facilitating the means of consultation in cases of difficulty and danger.

They have just published the first part of a volume of their Trans, actions, containing several important papers: the following is a list of them.—1. On the prevailing Diseases in St. Petersburgh, during the year 1819, by Blakus. 2. On the State of Ophthalmology in the Levant, by Milhausen. 3. On the Treatment of Croup by douches of cold water, by Harder and Muller. 4. Description of a remarkable Case of Exostosis of the Tibia, by Busch. 5. A remarkable Case of Pregnancy, by Wolff. 6. On the Diseases of the Tympanum, by Rauch. 7. On the Treatment of Venereal Complaints without Mercury, by Schmidt. 8. On Cold Affusion in Scarlet Fever, by Harder. 9. On the Treatment of Hydrophobia, by the same. 10. On the Mineral Waters of Italy, by Schmidt. 11, 12. Two practical Papers on Diseases of the Eye, by Lerche. 13. Case of Arthritis Anomala, by Harder. 14. On the good Effects of the Douches of Cold Water in Mental Alienation and Hypochondria, by Mylius. 15. New Experiments on Rabies. 16. Cure of Tetanus by Magnetism, by Weisse. Miscellanea. Meteorological Journal.

